# Conformational Variability in Ground-State CFTR Lipoprotein Particle Cryo-EM Ensembles

**DOI:** 10.3390/ijms23169248

**Published:** 2022-08-17

**Authors:** Luba A. Aleksandrov, Adrei A. Aleksandrov, Timothy J. Jensen, Joshua D. Strauss, Jonathan F. Fay

**Affiliations:** Biochemistry and Biophysics, University of North Carolina at Chapel Hill, 6107 Thurston Bowles Building, Chapel Hill, NC 27599, USA

**Keywords:** cryo-EM, CFTR, cystic fibrosis, electrophysiology, SMALP, lipids

## Abstract

Cystic fibrosis transmembrane regulator (CFTR) is a dynamic membrane protein belonging to the ABC transporter family. It is unusual within this family as it is an ion channel, as opposed to a transporter. Activation of CFTR requires ATP and phosphorylation by PKA, and dysregulation of CFTR mediated salt and water homeostasis can lead to cystic fibrosis. Recent advancements in structural biological methods have led to more than 10 published CFTR structures, and, so far, all of these structures of CFTR, determined by cryo-EM, have been limited to detergent-purified protein preparations. To visualize CFTR in an environment that more closely represents its native membranous environment, we utilized two different lipoprotein particle encapsulation techniques: one in which the ion channel is first purified and then reconstituted using the membrane scaffolding protein Saposin A and another that uses the solubilizing polymer Sokalan CP9 (DIBMA) to extract CFTR directly from membranes. Structures derived from these types of preparations may better correlate to their function, for instance, the single-channel measurements from membrane vesicles.

## 1. Introduction

Cystic fibrosis (CF) is a genetic disease affecting over 70,000 people globally and caused by mutations in the cystic fibrosis transmembrane regulator (CFTR) gene. The CFTR protein serves as an ion channel that plays a key role in salt and water homeostasis related to mucus hydration. The absence or impaired function of CFTR results in a transepithelial imbalance of ions and fluid in cells of the sweat glands, airways, intestine, and pancreas, among other organs. Although CF affects many organs, the respiratory aspect is a major cause of mortality, with mucus dehydration due to dysfunctional ion channel conductance leading to poor lung morbidity.

Triple combination therapy is a significant breakthrough in treating patients carrying the most common CF-causing mutation, ΔF508, among other variants, resulting in residual function. However, the atomic and molecular details that govern the dynamic process of regulation and channel-opening events of this cAMP-dependent, phosphorylation-activated anion channel have yet to be fully realized. Using cryogenic electron microscopy (cryo-EM), over 10 published structures of CFTR have been elucidated with nominal resolutions ranging from 2.7 to 6 Å [1,2,3,4,5,6].

The first CFTR structure to be determined was that of zebrafish CFTR (zfCFTR) [1]. Phylogenic analyses of CFTR from different species reveal that zfCFTR is one of the more divergent CFTR orthologues [7]. Interestingly, we find that zfCFTR is non-functional when expressed in a mammalian cell expression system, and its activity was measured using our established signal channel recording protocols. Confirming these findings, others also report zfCFTR as poorly functional, as it possesses an extremely low open-state probability and very low conductance [8]. Intriguingly, we observe that zfCFTR is architecturally extremely similar to published human CFTR (hCFTR) structures despite its lack of a clear significant wild-type, similar to hCFTR channel activity.

A comparison between the published atomic coordinates of zfCFTR and hCFTR shows a high degree of structural and conformational similarity, with the RMSDs between the structures for zfCFTR and hCFTR determined to be less than 2.5 Å for comparable states. A mutation of human equivalent residue E1371Q in the Walker B loop (also known as the EtoQ mutation) of nucleotide-binding domain 2 (NBD2) can create a channel that is deficient for ATP hydrolysis and, consequently, possesses a higher open-state probability [9]. Of note, the reported phosphorylated EtoQ mutant structures of hCFTR have been solved with CF potentiators [6] or correctors [10] bound. While the potentiators Ivacaftor and GLP1873 have been shown to enhance the open-state probability [11,12], the difference in the overall molecular architecture when bound is similar to the phosphorylated zfCFTR.

This raises some interesting questions about the structural and functional state of hCFTR. Potentiators should increase the open-state probability, yet it appears they are bound to an inactive-like state similar to that of zfCFTR, which is poorly functional. Moreover, the EtoQ mutation should also increase the propensity of an open-like state, yet what is observed is a structure with a striking similarity to the inactive zfCFTR structures.

These structures and others were determined in the presence of the detergent digitonin [1,2,3,4,6,10]. The hydrophobic core of a digitonin micelle is thought to be solid in comparison to the fluid interior of most other micelles, which has been brilliantly utilized in a number of cryo-EM membrane protein structures (especially in the ABC family of proteins) in the last few years. However, digitonin’s ability to restrict the conformational mobility of the protein—which is highly desirable for structural studies—could limit certain key physiological end points. It follows, then, that these important physiological states may be more difficult to observe in systems using digitonin as opposed to lipids.

Digitonin has been shown to thermostabilize rhodopsin compared to other detergents [13], and conformational transition to an active state has been extensively studied [14,15,16], suggesting that digitonin preparations stabilize a less active intermediate, as compared to more permissive detergent and/or lipid preparations. Similar findings have been observed for ABC transporters in nanodisc lipoprotein particle preparations compared to other ABC proteins in digitonin. In these studies, the outward-facing open conformation is more readily observed in lipid preparations [17] in contrast to the outward-facing occluded conformations that have dominated digitonin ABC cryo-EM protein models [18,19]. It has been known for some time that ATP turn-over for ABC transporters can be enhanced in either mixed lipid–detergent micelles or nanodiscs [20], providing further evidence that the membrane environment can modulate protein function.

Here, we explore the use of two lipoprotein particle reconstitution methods for biochemical characterization and biophysical analysis of avian CFTR. We find that using either a saposin-lipoprotein nanoparticle (Salipro) [21] or Diisobutylene-maleic acid (DIBMA, also known as Sokalan CP9) lipoprotein particle appears to be more permissive and enhances the conformational landscape of CFTR in comparison to digitonin detergent-solubilized CFTR preparations.

## 2. Results

**Figure 1:**
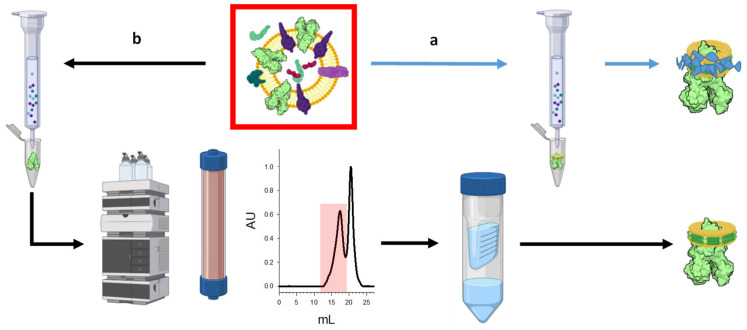
Two methods for generating CFTR lipid nanoparticles from CFTR-containing membranes (red box): (a) a rapid, one-step process of direct solubilization using DIBMA and (b) the two-step process of detergent purification and lipoprotein particle reconstitution using Saposin A.

For these studies, we use our previously described, inherently more thermostable chicken CFTR construct ΔRI/1404S/1441X, which we name TSch (thermostable-chicken CFTR) [4]. We previously found the avian orthologue to be more thermally stable than human CFTR and, when containing the H-loop mutant 1404S, we also observe an enhanced Po compared to Wt [4,22]. Here, we use BHK cells expressing TSch and purified TSch in the presence of Mg and ATP, as we have found that with these conditions the channel is predominately inactive and can become active after treatment with PKA. The first approach utilized a DIBMA polymer to generate detergent naïve lipoprotein particle preparations (Figure 1a), allowing CFTR to be solubilized directly from the membranes. Unlike in our implementation of the Salipro method, the protein was not first purified in detergent and reconstituted into lipids. Since detergent solubilization was never performed, this approach might arguably reveal a less adulterated native CFTR structure in a membrane environment. The second approach employed detergent-purified TSch, as previously described [4], and then subsequent incorporation into Saposin A-derived lipid nanoparticles, termed Salipro [21] (Figure 1b).

### 2.1. DIBMA–TSch Assembly and Cryo-EM Analysis

**Figure 2:**
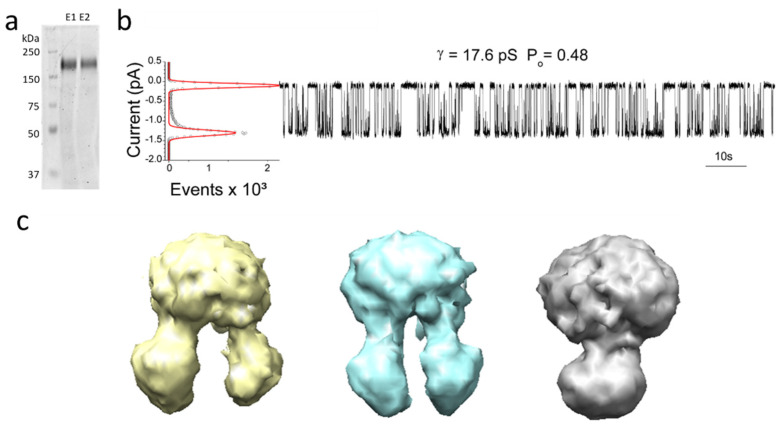
Analysis of TSch CFTR in a DIBMA lipoprotein particle (DIBMA-TSch): (**a**) SDS-PAGE analysis of elution fractions showing DIBMA-solubilized TSch can be purified. (**b**) Elution fractions from A applied to bilayers and single-channel recording of PKA-treated purified CFTR were measured. All-points histogram used to calculate Po is shown on the left of the upper recording (Po = 0.48). (**c**) The cryoSPARC-derived ab initio models generated from 50 k 2D-classified particles within a 2000-micrograph data set. Imaging conditions were 1.07 Å/pixel with a total dose of (~60 e^−^/Å^2^) on a K3 direct electron detector.

To elucidate the lipid structure of CFTR, we employed the polymer DIBMA (Sokalan CP9), which has been used to directly solubilize integral membrane proteins in their host membranes. DIBMA was used to solubilize naïve TSch from BHK membranes and immobilized metal affinity chromatography (IMAC) produced purified, detergent-free CFTR containing lipoprotein particles (Figure 2a).

The PKA-treated lipoprotein particles were then applied to planar lipid bilayers and the DIBMA–TSch lipoprotein particle was spontaneously fused. Interestingly, the open-state probability (Po) for the PKA-treated CFTR isolated under these conditions was 0.48 (Figure 2b) at 37 °C, which is lower than the typically observed nearly locked open state with a Po of 0.96 for TSch in BHK microsomes fused to the same lipid bilayer [4]. It is possible that the local lipid content in the ‘annular shell’ for DIBMA–TSch would be enriched in these preparations compared to microsomes. Other polymer lipoprotein particle encapsulation studies observed impaired activation [23], and these results further suggest that the local lipid microenvironment could alter channel activation.

Purified functional DIBMA–TSch ion channels were then applied to Quantifoil grids and imaged using a 200 keV Talos Arctica. Although a secondary structure was not apparent in the initial 2D classification, which typically indicates a low-resolution structure, the 3D class averages could produce 15–20 Å reconstructions for this data set. Initial ab initio models from the selected 2D classes suggest the possibility of multiple conformations present within the DIBMA–TSch cryo-EM ensemble (Figure 2c). These findings suggest that perhaps the enhanced conformational heterogeneity of states could be limiting high-resolution structure maps in the smaller data sets of DIMBA-TSch.

### 2.2. Salipro–TSch Assembly, Function, and Cryo-EM Analysis

**Figure 3:**
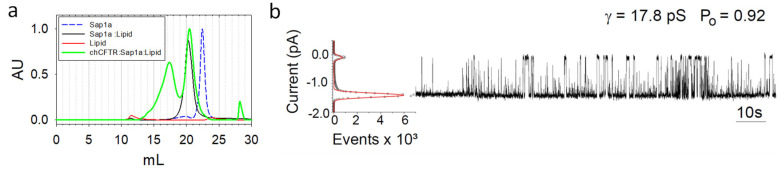
Analysis of TSch CFTR in a Saposin lipoprotein particle (Salipro-TSch). (**a**) Size exclusion chromatography (SEC) analysis of Saposin A after incubation with CFTR and lipids (green); also shown are Saposin A alone (blue dash), lipids alone (red), and empty lipoparticles (black). Samples were run on two Shodex KW-804 elution profiles monitored by tryptophan fluorescence (excitation 290 nm, emission 340 nm). (**b**) CFTR lipoprotein particle from the first peak fractions (~16–18 mL retention fractions) from A were concentrated and applied to bilayers, and single-channel recordings of PKA-treated purified CFTR were measured. The all-points histogram used to calculate Po is shown on the left of the upper recording (Po = 0.92).

Membranes containing ground-state TSch (i.e., not treated with PKA) were solubilized in DMNG and eluted from IMAC columns in digitonin, as previously described [4], and then the digitonin–TSch was reconstituted with Saposin A and lipids (3:1 mix of DOPS and DOPG) at a ratio of 0.5:1:1 [protein:Sap1a:Lipids]. Monomeric CFTR in a Saposin–lipid nanoparticle (Figure 3a) and CFTR-incorporated vs. -unincorporated lipoparticles were then separated by size exclusion chromatography. Note that the first green peak in Figure 3a has CFTR-containing lipid nanoparticles (also confirmed by a Western blot analysis of fractions and Coomasie-stained SDS-PAGE of the concentrated peaks). The second peak that appears at around a 20 mL elution volume corresponds to free (or empty) lipoparticles. A control run of the Saposin and lipids only (Figure 3a, black) shows the presence of free non-CFTR-containing lipoparticles, which has a similar retention time to the non-CFTR-containing lipoprotein particle peak (second peak in green). An immuno-blot analysis of the fractions also confirmed the absence of CFTR in this second green peak. A third minor peak that elutes at a volume much smaller than the that of free Saposin (11 kDa, blue dash) near the extremely low molecular weight range (~27 mLs) likely represents imidazole present in the CFTR elution fraction that was not removed prior to the reconstitutions, as we found this to be an unnecessary step.

These purified, detergent-liberated lipoprotein particle complexes were then tested for their ability to function via single-channel recordings. Importantly, we indeed do find similar functional properties for the purified, reconstituted lipoprotein particle preparations that are identical to the properties seen in the BHK membranes from which they are expressed (Figure 3b compared to [4]).

Of note, the initial studies that applied PKA-treated Salipro–TSch to planar lipid bilayers were not successful. As an alternative, Salipro–TSch was incubated with lipids (destabilized with Triton liposomes), detergent, and SM-2 Biobeads to generate proteoliposomes. These TSch-containing proteoliposomes behaved similarly to those of previous findings with an open-state probability (Po) of 0.92. Our results suggest that purified, reconstituted Salipro–TSch retains functional channels after purification, incorporation into lipoprotein particles, and subsequent reconstitution into proteoliposomes, as previously shown for digitonin–TSch.

The lipoprotein particles containing CFTR samples were then subjected to multiple screening sessions on a 200 keV electron microscope to identify optimal imaging conditions as a function of lipids used in the formation of the nanoparticles. The best of these initial screening sessions was then imaged using a 300 keV Titan Krios. The peak containing Salipro–TSch was concentrated to ~0.2 mg/mL and applied to Quantifoil Holey Carbon grids.

### 2.3. Structure Analysis

**Figure 4:**
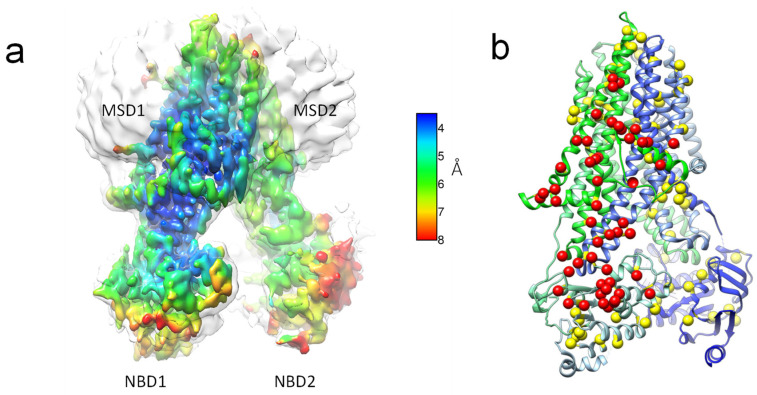
Analysis of CFTR in a Salipro–lipoprotein nanoparticle. (**a**) Local resolution heat-map plotted to the surface of Saposin-derived TSch CFTR 4.22 Å cryo-EM map, illustrating clear secondary structure in the consensus map. Note the region colored blue in MSD1, and the inner core of NBD1 is more well-resolved, with apparent side chain densities. (**b**) Red and yellow balls show location of CFTR2 database point mutations (https://cftr2.org/, accessed on 21 January 2022) mapped to a model human CFTR (5UAK; [2]). Note MSD1 and inner core of NBD1 on the left side (shown as red spheres) have more point mutations than the other half of the protein (MSD2 and NBD2) on the right. About 60% of known point mutations appear to coincide with the more well-resolved region of MSD1/NBD1 that we observe for CFTR when reconstituted in a lipid nanoparticle.

Interestingly, the predominate Salipro–TSch reconstruction that we observe resolves to around 4.22 Å by gold-standard Fourier shell correlation (Figure 4). Overall, the architecture is more similar to the ATP-free dephosphorylated zebrafish CFTR (apo-zfCFTR) structure than our previous digitonin-solubilized TSch (Digi-TSch) derived cryo-EM models [1,4]. The NBD separation is further apart than our previous Digi–TSch and again resembles apo-zfCFTR, and the relative transmembrane helical (TMH) orientation appears essentially similar to that of apo-zfCFTR. At the current resolution, there is some ambiguity regarding the locations of TMH8 and TMH5, but this could suggest a conformational mobility of this region. Our reconstruction appears to be similar to intermediate reconstructions of the EMPAIR-10281 data set in the TMH8 and TMH5 regions, suggesting that extensive data collection is likely to produce a reconstruction in which this region is the same as previous structures [1,2,3,5,6,10]. The alternative location of TMH7 and “straight TMH8” that we previously observed in our digitonin–TSch [4] does not appear to exist in our Salipro–TSch cryo-EM reconstructions. This is supported by the observation of a low-resolution density consistent with glycosylation of extracellular loop 4 (EL4) in a position that is congruous with the apo-zfCFTR helical locations of TMH7 and 8.

Additionally, we observe a lower resolution and less coherent density for NBD2 in our reconstruction, suggesting that NBD2 is perhaps more mobile in the Salipro–TSch cryo-EM ensemble. A 3D variability analysis in cryoSPARC also corroborates the dynamic nature of MSD2 and NBD2. As NBD2 is connected to TMH8 via a coupling helix, it may be possible that this mobility acts to transmit conformational mobility to the upper portion of TMH8, which is difficult to clearly observe in our current data set. The overall dynamics of the ensemble are also evidenced in the local resolution (Figure 4a), which shows a higher local resolution membrane-spanning domain 1 (MSD1) over MSD2, suggesting that MSD1 is more stable than MSD2. The higher local resolution of MSD1 was previously observed in most digitonin-solubilized CFTR reconstructions (though less extreme than in these Salipro-lipoparticle preparations), with an ordered sterol-like density surrounding the extracellular portions of the MSD1 helices and the lasso region interactions on the inner membrane boundary likely contributing to this stability. As 3D reconstructions also appear to have a similar rigidity of MSD1 over MSD2 [1,3,4,5], this possibly suggests a potential mechanism by which the conformational flexibility of MSD2 could be induced by changes in the NBD2 dynamics. At low contour values, we also observe more membrane irregularities around the TMH7 and TMH8 regions at the Saposin/lipid boundary.

## 3. Discussion

Here, we present our cryo-EM results for ground-state CFTR from two different lipoprotein particle encapsulation methodologies. Our analyses demonstrate that functional CFTR in lipids can be incorporated into planer bilayers and channel activity recorded. DIBMA-encapsulated lipoprotein particles also retain function and have a rapid and straightforward purification protocol. Analysis of a small micrograph data set suggests that there are also global protein dynamics within these alternative detergent, naïve DIBMA microenvironments (Figure 3). In other words, the protein ensemble appears to have more varied conformational states when plunge-frozen. In the more well-resolved Salipro–lipoprotein particle preparations, membrane-spanning domain 1 (MSD1) appears more ordered than MSD2, as clearly demonstrated by the local resolution plotted to the surface as a heat map (Figure 4a). The higher local resolution of MSD1 is consistent with previous cryo-EM maps in the detergent digitonin; however, the magnitude of the local resolution variation between MSD1 and MSD2 appears to be even more extreme in our Salipro-encapsulated TSch CFTR. Known CFTR disease mutations appear to coincide with this more well-resolved region of MSD1 that we observed for CFTR when reconstituted in a lipid nanoparticle (Figure 4b). Thus, the stability of this MSD1/NBD1 is important for proper protein function, and mutations within this region can lead to dysregulation and CF.

In contrast to the enthalpic stability of the MSD1/NBD1 of CFTR, the MSD2/NBD2 appears to have greater conformational entropy. CryoSPARC 3D variability analysis (3DVAR) also supports the inference of the structural dynamics of MSD2/NBD2 in the cryo-EM Salipro–TSch–lipoprotein particle ensemble (Supplementary Video S1). Intermediate-state 3DVAR analysis appears to couple NBD2 mobility with conformational changes in MSD2, specifically around TMH5, 7, and 8. Thus, as NBD2 moves closer to NBD1, the extracellular regions of TMH5, 7, and 8 become less well-resolved due to increased conformational dynamics. This suggests a greater heterogeneity within these regions for Salipro–TSch particles in the cryo-EM ensemble. RosettaCM was also employed to gain insight into the conformational variability of the Salipro–TSch ensemble [24]. We observe a greater divergence of NBD2, as well as the extracellular regions of TSch and the solvent exposed region of NBD1 between the top 10 RosettaCM models built into the consensus map (Figure S1). These observations and analyses provide insight to a potential mechanism for allosteric coupling between NBD2 and the extracellular regions of TMH5, 7, and 8 that could have a role in channel gating. In support of this notion, deletion of NBD2 can produce productive PKA-dependent channel events, although with a much lower Po than that of Wt CFTR [25].

**Figure 5:**
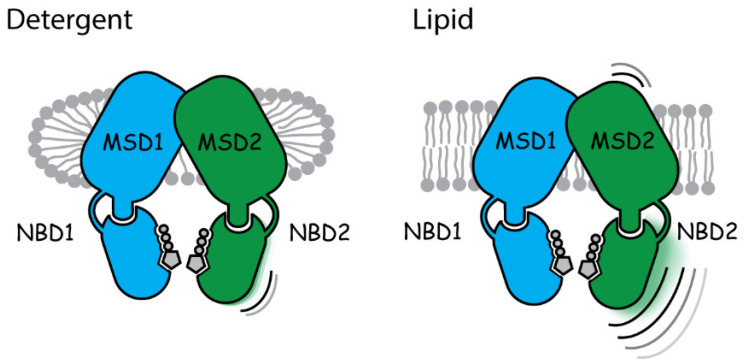
Cartoon illustrating environmental effects on CFTR structure. Increases in the conformational dynamics of MSD2 and NBD2 can be observed in lipid nanoparticles containing CFTR (Salipro–TSch) compared to their detergent (digitonin–TSch) counterpart. See Supplementary Video S1 for more details.

Structures derived from these lipoprotein preparations suggest the non-PKA-treated ground-state of CFTR can exist in varying degrees of nucleotide-binding domain separation and transmembrane helical transitions in the extracellular portions of MSD2. In conclusion, our studies suggest that the lipid environment can increase the conformational heterogeneity of the inactive CFTR ground-state structures compared to their detergent digitonin-solubilized counterparts (Figure 5).

The potentiators Ivacaftor and GLP1837 have been observed binding to MSD2 inside the membrane [6] around the more variable region of TMH8, while the correctors Lumacaftor and Tezacaftor have been observed binding inside the membrane to the more well-ordered region of MSD1 [10]. Notably, the potentiators appear to bind to the region around TMH7/8 that is the least well-resolved in our reconstructions and, thus, arguably represents a highly dynamic region within MSD2.

Therefore, it is possible that the conformational dynamic regions of CFTR can be exploited as regions for targeting small molecule therapeutics. Additionally, the MSD1 transmembrane helical bundle with an observed more ordered structure may become destabilized by CF mutations, which seem to cluster in this area; thus, correctors that bind near to this stable region may function by enthalpically stabilizing this well-ordered core. Interestingly, for ATP-sensitive potassium (KATP) channels, the binding site for KATP chaperones and channel inhibitors (glibenclamide, repaglinide, and carbamazepine) appear to bind to a common pocket on MSD1 between the two halves of the sulfonylurea receptor 1 (SUR1) subunit [26,27]. The densities assigned to Tezacaftor and Lumacaftor have been observed in the CFTR on MSD1 in the well-ordered region of MSD1, specifically, TMH2, TMH6, and the elbow portion of TMH1. This corresponds to a region on the ‘outside’ of MSD1 facing the membrane, as opposed to the cytoplasmic exposed ‘inside’ region observed for SUR1 bound ligands (see Figure S2 for more details). These two binding sites on either side of TMH3, between the two ABCC family proteins, engage this well-ordered region of MSD1 through different mechanisms. These observations suggest that the rigid MSD1 region in the ABCC family is a potential hotspot for pharmacological manipulation and chaperone binding.

Continued progress and support of research that examines the conformational heterogeneity of CFTR in various lipid environments is crucial for correct interpretations of the structural and functional relationships in a physiologically relevant setting. Moreover, we look forward to the experimental release of 3D Flexible Refinement in cryoSPARC [28] to help extract greater molecular clarity for these dynamic CFTR–lipoprotein particle-encapsulated cryo-EM ensembles. With the elucidation of new intermediates and existing structures, there is potential to help guided drug-discovery efforts in the search for that next-generation of CF therapeutics targeting structurally homogenous regions as well as more dynamic heterogenous regions.

## 4. Materials and Methods

### 4.1. Expression and Purification of ABCC7

Expression of the chicken ΔRI/1404S/1441X/His CFTR (TSch) construct was performed as previously described [4]. Detergent purification was essentially as previously described [4,22]; in brief, 100 mg (determined by Bradford) of cell membranes yielded 300 μg of detergent-purified TSch. Membranes were first solubilized with 1% DMNG applied to Co-Talon resin (Clontec) and eluted with imizidole in buffer A [40 mM Tris–HCl, pH 7.4; 0.15 M NaCl; 5 mM ATP-Mg; 10% glycerol, 0.06% digitonin]. Eluates at a CFTR concentration ~0.5–0.7 mg/mL were directly used for Salipro nanoparticles reconstitution.

### 4.2. Incorporation of the Purified TSch into Salipro Nanoparticles

Lipids (DOPC/DOPS at 3:1 ratio) solubilized in DDM buffer were incubated for 5 min at 37 °C, and then purified TSch was added at a molar ratio 1:2 (TSch/lipid) and incubated for 15 min at toom temperature. Saposin A was purified from E. coli Rosetta gami-2 (DE3) (Novagen, Madison, WI, USA), as previously described [21]. Purified Saposin A was added to the lipid–protein mixture (CFTR/lipid/Saposin–molar ration 0.5:1:1) and incubated for another 10 min at room temperature; the mixture was quickly diluted about 5-fold with detergent-free buffer B (40 mM Tris-HCl pH 7.5, 150 mM NaCl, 5 mM Mg-ATP). The sample was subjected to a gel-filtration step on a Superdex S200 analytical gel filtration column, equilibrated, and eluted with buffer B. Fractions containing Salipro–TSch were pooled, concentrated to around 0.2–0.5 mg/mL using Amicon Centricon filter devices, and used for biochemical and structural studies.

For single-channel measurements, the Salipro–TSch sample was reconstituted into the liposomes, as previously described [22]. Briefly, Salipro–TSch was incubated with Triton X-100 destabilized liposomes (DOPE/DOPC/DOPS/Cholesterol; 48:22:12:18) at a 1:60 (*w*/*w*) protein-to-lipid ratio and incubated for 30 min at 4 °C with gentle agitation. Traces of detergent were removed by incubation with Bio-Beads SM2.

### 4.3. Membranes Solubilization in DIBMA

Membranes from the BHK-21 cells expressing TSch (ΔRI/1404S-1441X/His) were resuspended in buffer C (40 mM Tris-HCl, pH-7.5; 500 mM NaCl; 5 mM MgCl 2; 5 mM ATP; 15% glycerol; protease inhibitor cocktail: benzamidine at 120 μg/mL, E-64 [trans-epoxysuccinyl-l-leucylamido-(4-guanido)butane] at 3.5 μg/mL, aprotinin at 2 μg/mL, leupeptin at 1 μg/mL, and Pefabloc at 50 μg/mL), and then DIBMA polymer was added to final concentration of 1%. After 15 min incubation at 4 °C, the sample was spun at 100,000× *g* for 30 min, and supernatant was discarded. The stripped membranes were resuspended in the same buffer and solubilized with 2.5% DIBMA with gentle shaking for o/n at 4 °C. Insoluble material was removed by centrifugation (100,000× *g* for 30 min).

### 4.4. DIBMA–TSch Particles Purification

The supernatant was mixed with Co-Talon agarose (Clontech, Palo Alto, CA, USA) and gently rotated overnight at 4°C. The bound resin was washed with buffer A and CFTR eluted with 100 mM imidazole. Elution fractions were then analyzed using 7.5% SDS–PAGE and InstantBlue stain (Expedeon, Cambridge, MA, USA). Samples containing the DIBMA–TSch–lipoprotein particles after buffer exchange with Zeba desalting columns (Thermo Scientific, Waltham, MA, USA) were used directly for the single channel measurements and cryo-EM.

### 4.5. Single-Channel Measurements

The detailed protocol of the CFTR single-channel recording and data analysis was performed as previously described [4]. Briefly, planar lipid bilayers were prepared by painting a 0.2 mm hole drilled in a Teflon cup with a phospholipid solution in n-decane containing a 3:1:1 mixture of 1-palmitoyl-2-oleoyl-sn-glycero-3-phosphoethanolamine, 1-palmitoyl-2-oleoyl-sn-glycero-3-phosphoserine, and cholesterol (Avanti Polar Lipids, Alabaster, AL, USA). The lipid bilayer separated 1.0 mL of a solution in the Teflon cup (“cis” side) from 5.0 mL of a solution in an outer glass chamber (“trans” side). Both chambers were magnetically stirred and thermally insulated. Heating and temperature control were established by a temperature control system (TC2BIP, Cell Micro Controls, Norfolk, VA, USA).

CFTR ion channels were transferred into the preformed lipid bilayer by a spontaneous fusion of lipoprotein particles containing DIBMA–TSch or Salipro–TSch reconstituted into the liposomes. All measurements were done in symmetrical salt solution (300 mM Tris–HCl, pH 7.2, 5 mM MgCl2, and 1 mM EGTA) under voltage-clamp conditions by using an Axopatch 200B amplifier. The single-channel current was filtrated by Low Pass Bessel filter with a cut off frequency of 50 Hz, digitized by Digidata 1322 (Molecular Devices, LLC., Sunnyvale, CA, USA) with a sampling rate of 500 Hz, and analyzed using pCLAMP 9.2 software (Molecular Devices, LLC, Sunnyvale, CA, USA). Origin 7.5 (OriginLab Corp., Northampton, MA, USA) was used to fit all-point histograms by multi-peak Gaussians. A single-channel current was defined as the distance between peaks on the fitting curve and was used for calculation of the single-channel conductance. The single-channel open probability (Po) was calculated as the ratio of the area under the peak for the open state to the total area under both peaks on the fitting curve.

### 4.6. Cryo-EM Sample Preparation for Salipro–TSch

Ice-cold, purified Salipro–TSch at 0.2 mg/mL in EM sample buffer was added (5 μL) to glow discharged grids (medium setting, glow time 1 min) using a Harrick PDC-32 g plasma cleaner. Quantifoil R1.2/1.3 400 mesh Au Holey Carbon grids were blotted (3 s) with Vitrobot Mark IV (FEI) and plunge-frozen in liquid ethane. The Salipro–TSch data set consisted of 4671 images collected from two different grids, and the data collection sessions were automatically collected via Latitude using a 300 keV FEI Titan Krios with a Gatan K3 direct electron detector. Images were recorded at a physical pixel size of 1.07 Å for ~2.6 or 4.2 s at ~15 electrons/pixel/second divided into 100 frames or 60 frames to give an exposure dose of ~39 or 64 electrons per Å^2^, respectively. The first set required a lower exposure as the ice appeared to melt during initial image acquisition, whereas the second set did not have this issue. The micrographs had a calculated mean defocus of 1.9 μm (SD = 0.8 μm) under focus positive. Micrograph alignments were performed using MotionCor2 [29] after unzipping, unpacking, and conversion from dm4 to gain a corrected, normalized mrc format. All subsequent CTF corrections, two-dimensional or three-dimensional classifications, and refinement steps were performed using cryoSPARC or RELION [30,31]. Initial particles were selected using cryoSPARC blob particle picker, and the initial 2D classification yielded templates for subsequent template picking.

Manual curation divided the data into three groups termed: “good”, “bad”, and “ugly”. The good group was defined as decent micrographs sufficient for autopicking; bad micrographs were discarded (i.e., carbon, black ice, etc.), and the ugly set contained micrographs that were usable but would likely require more manual intervention to ensure a high particle-pick fidelity (i.e., around 30% or more of the images containing carbon or other aberrations occurring due to poor shot alignment). Autopicking was performed on good and ugly sets using combined blob picking via cryoSPARC and DoGpicker [31,32]. The ugly set, which suffered from a high degree of ‘autopicks’ on carbon was manually deselected in RELION [30]. Several rounds of 2D classification in either cryoSPARC or RELION were performed to eliminate false positives. Template picking was then implemented on the curated set using class averages and then combined with the surviving particles from blob picking and 2D classification. The unique set of 347,273 particles was then subjected to multiple rounds of 3D classification to retain 84,564 particles in the final data set that resolved to a nominal resolution of 4.22 Å using non-uniform 3D refinement [33] (by FSC using the 0.143 Å cut-off criterion) [34]. Post-processing, including soft masking, B-factor sharpening in cryoSPARC, and filtering by local resolution [35], and post sharpening were performed on the two half-maps using deepEMhancer [36]. The consensus map was used in conjunction with Rosetta Comparative Modeling (RosettaCM) to generate 1000 models using hCFTR (5UAR) as a template model, and the top 10 models were selected based on lowest elec_dens scores [24].

### 4.7. Cryo-EM Sample Preparation for DIBMA–TSch

Ice-cold, purified DIBMA–TSch at 0.2 mg/mL in EM sample buffer was added (5 μL) to glow discharged grids, as described above. The DIBMA–TSch data set consisted of 2249 images collected from two different grids, and data collection sessions were automatically collected via SerialEM [37] using a 200 keV FEI Talos Arctica with a Gatan K3 direct electron detector. Images were recorded at a physical pixel size of 0.91 Å for 60 frames to give an exposure dose of ~63 electrons per Å^2^. Autopicking was performed as described above for Gaussian blobs, and 2D classification and ab initio models were generated using cryoSPARC [31].

## Data Availability

The cryo-EM map of Salipro–TSch has been deposited to EMDB.

## Supplementary Materials

The following supporting information can be downloaded at: https://www.mdpi.com/article/10.3390/ijms23169248/s1.
