# Supplementary material for: Conformational Variability in Ground-State CFTR Lipoprotein Particle Cryo-EM Ensembles
Source: Int J Mol Sci. 2022 Aug 17;23(16):9248. doi: 10.3390/ijms23169248 (PMC9409475; doi:10.3390/ijms23169248)
Supplement: Supplementary file 1 [file ijms-23-09248-s001.zip › ijms-1768642-supplementary.pdf]

Supplementary Figures

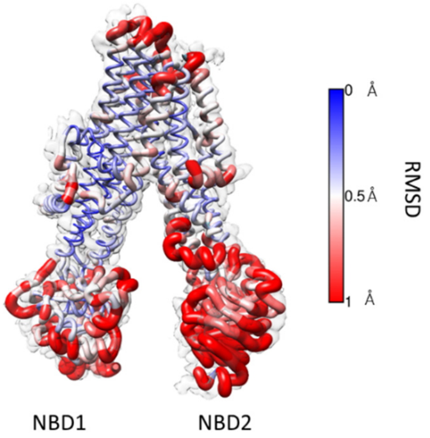

Figure S1. Root mean squared deviation (RMSD) at Cα for the top 10 Rosetta refinement models against the globally refined Salipro–TSch map (translucent). The RMSD values are colored from blue to red (0-1Å). In addition, the thickness of the wire/tube representation correlates with the level of RMSD. See Materials and Methods for more details. Note NBD2, intracellular loop two, the solvent exposed region of NBD1, and extracellular loops appear to have the most deviation between models.

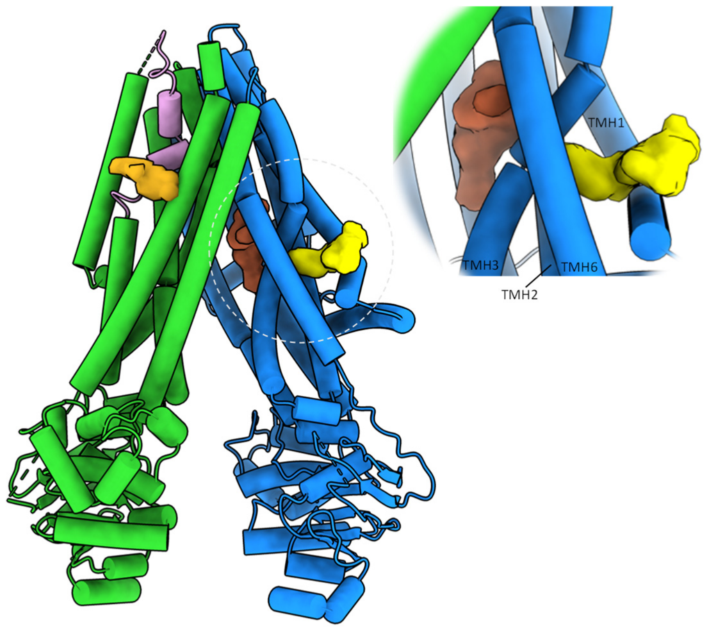

Figure S2. **Location of ligand-binding sites for ABCC family members CFTR and KATP.** Binding site location of the CFTR potentiators Ivacaftor and GLP1837 (orange) on the outside of MSD2 (green) near the upper 'kinked' part of TMH8 (orcid). On the outside of MSD1 (blue) is the binding site location of Tezacaftor and Lumacaftor (yellow) and on the inside of MSD1, directly across from TMH3, is the binding sites of SUR1 ligands glibenclamide and repaglinide (coral) aligned to CFTR. Inset, shows the location of a hot-spot (dotted gray circle) for stabilization of ABCC proteins by small molecule therapeutics.

Table S1. Cryo-EM data collection and validation statistics for Saliprot–TSch.

Magnification	81,000
Pixel size (Å)	1.07
Voltage (kV)	300
Electron exposure (e–/Å^2)	39 & 64
Number of movies used	4671
Defocus mean (SD) µm[1]	1.9 (0.8)
Range	0.4–5.0
Symmetry imposed	C1
Initial particle images (no.)	347,273
Final particle images (no.)	84,564
Map resolution (Å)	2.45
FSC threshold	0.143
Bfactor	172
Map resolution range (Å)	3.5–8.8
